# Statins Increase Plasminogen Activator Inhibitor Type 1 Gene Transcription through a Pregnane X Receptor Regulated Element

**DOI:** 10.1371/journal.pone.0138097

**Published:** 2015-09-17

**Authors:** Frederick M. Stanley, Kathryn M. Linder, Timothy J. Cardozo

**Affiliations:** 1 Department of Biochemistry and Molecular Pharmacology, New York University School of Medicine, New York, New York, United States of America; 2 Laura and Isaac Perlmutter Cancer Center, NYU Langone Medical Center, New York, New York, United States of America; University of Texas Health Science Center, UNITED STATES

## Abstract

Plasminogen activator inhibitor type 1 (PAI-1) is a multifunctional protein that has important roles in inflammation and wound healing. Its aberrant regulation may contribute to many disease processes such as heart disease. The PAI-1 promoter is responsive to multiple inputs including cytokines, growth factors, steroids and oxidative stress. The statin drugs, atorvastatin, mevastatin and rosuvastatin, increased basal and stimulated expression of the PAI-1 promoter 3-fold. A statin-responsive, nuclear hormone response element was previously identified in the PAI-1 promoter, but it was incompletely characterized. We characterized this direct repeat (DR) of AGGTCA with a 3-nucleotide spacer at -269/-255 using deletion and directed mutagenesis. Deletion or mutation of this element increased basal transcription from the promoter suggesting that it repressed PAI-1 transcription in the unliganded state. The half-site spacing and the ligand specificity suggested that this might be a pregnane X receptor (PXR) responsive element. Computational molecular docking showed that atorvastatin, mevastatin and rosuvastatin were structurally compatible with the PXR ligand-binding pocket in its agonist conformation. Experiments with Gal4 DNA binding domain fusion proteins showed that Gal4-PXR was activated by statins while other DR + 3 binding nuclear receptor fusions were not. Overexpression of PXR further enhanced PAI-1 transcription in response to statins. Finally, ChIP experiments using Halo-tagged PXR and RXR demonstrated that both components of the PXR-RXR heterodimer bound to this region of the PAI-1 promoter.

## Introduction

PAI-1 inhibits dissolution of clots by its action on tissue type and urokinase plasminogen activators [1,2]. It also inhibits cell migration through its effects on the urokinase-type plasminogen activator receptor and integrin [3]. These dual roles lead to the many seemingly contradictory effects of PAI-1. For example, PAI-1 knockout mice recovered more slowly than wild type mice after myocardial infarction [4], but transgenic overexpression of PAI-1 in arterial endothelial cells resulted in cardiac occlusion [5]. This contradiction can be explained if PAI-1 is acutely necessary for wound repair, but its chronic expression is harmful due to increased fibrosis. Thus, the precise regulation of PAI-1 is critical and its overexpression in diabetes and other inflammatory states is associated with heart disease [6] and other complications [7]. Most, if not all, cell types produce PAI-1 in response to stress. Regulation is at the transcriptional level since PAI-1 is not stored and is rapidly inactivated after release into the blood. Numerous transcription factors were shown to activate PAI-1 expression including TGFß, glucocorticoids, HIF-1, AP-1, SP1 and FoxO3a [8–13].

The first nuclear receptors to be identified were the receptors for the steroids and thyroid hormone. Molecular cloning subsequently identified many related family members [14]. The nuclear receptors have a common domain structure characterized by the N-terminal A/B domain, the zinc finger DNA-binding domain (DBD or C), a short spacer sequence (D), the leucine zipper ligand-binding domain (E or LBD) and the C-terminal (F) domain. Transcriptional activation regions are localized to the center of the A/B domain and helix 12 of the LBD. Nuclear receptors bind to direct or inverted repeats of the sequence AGGTCA with various lengths of spacer DNA [15]. For example, pregnane X receptor (PXR) and vitamin D receptor (VDR) bind to a DR + 3 (AGGTCANNNAGGTCA). Nuclear receptors function by recruiting corepressors and coactivators to the promoter. Thus, corepressors such as NCoR that bind the unliganded thyroid hormone receptor repress the promoter. The ligand, triiodothyronine, acts as a switch that releases the corepressor and recruits coactivators such as steroid receptor coactivator 1 (SRC1) to increase transcription.

Nuclear receptors probably evolved to sense and regulate metabolite availability [16–18] and the first ligands were likely lipid metabolites. Thus, the oxysterols are well-defined ligands for liver X receptor (LXR) and they function to increase the availability of critical metabolic intermediates. These receptors were adapted for intracellular signaling (hormones) during the evolution of the metazoans. This hypothesis is supported by the presence of lipids in the binding cavity of nuclear receptors that were crystalized using bacterially expressed proteins. Studies showing the activation of classical steroid/thyroid receptors by farnesyl pyrophosphate support this hypothesis [19].

Statin drugs were developed to lower high levels of cholesterol that are a risk factor for heart disease by inhibiting the rate-limiting enzyme in cholesterol biosynthesis, HMG-CoA reductase. However, inhibition of this early step in cholesterol biosynthesis in turn reduces all of the metabolic intermediates of cholesterol biosynthesis. This includes such important molecules as farnesyl pyrophosphate and geranylgeranyl pyrophosphate, key players for prenylation and localization of signaling molecules such as Ras and Rho [20,21]. It also includes the isoprene moieties that are necessary for coenzyme Q that is crucial for oxidative phosphorylation [22]. The changes in a cell’s lipid concentration due to statin activity may increase or decrease endogenous ligands/modifiers for nuclear receptors [23]. Additionally, statins are close homologs of the lipid metabolites that they inhibit, and thus statins may be nuclear receptor ligands [24]

We found that statins increased PAI-1-luciferase (PAI-1-Luc) reporter activity in several cell types despite a prior study that described the contrary [25]. We confirmed our findings using RT-qPCR and identified a DR + 3 nuclear receptor response element in the PAI-1 promoter. Here we report these findings along with extensive analysis of this element suggesting that it is a PXR responsive site.

## Results

### Statins Activate the PAI-1 Promoter

**Fig 1:**
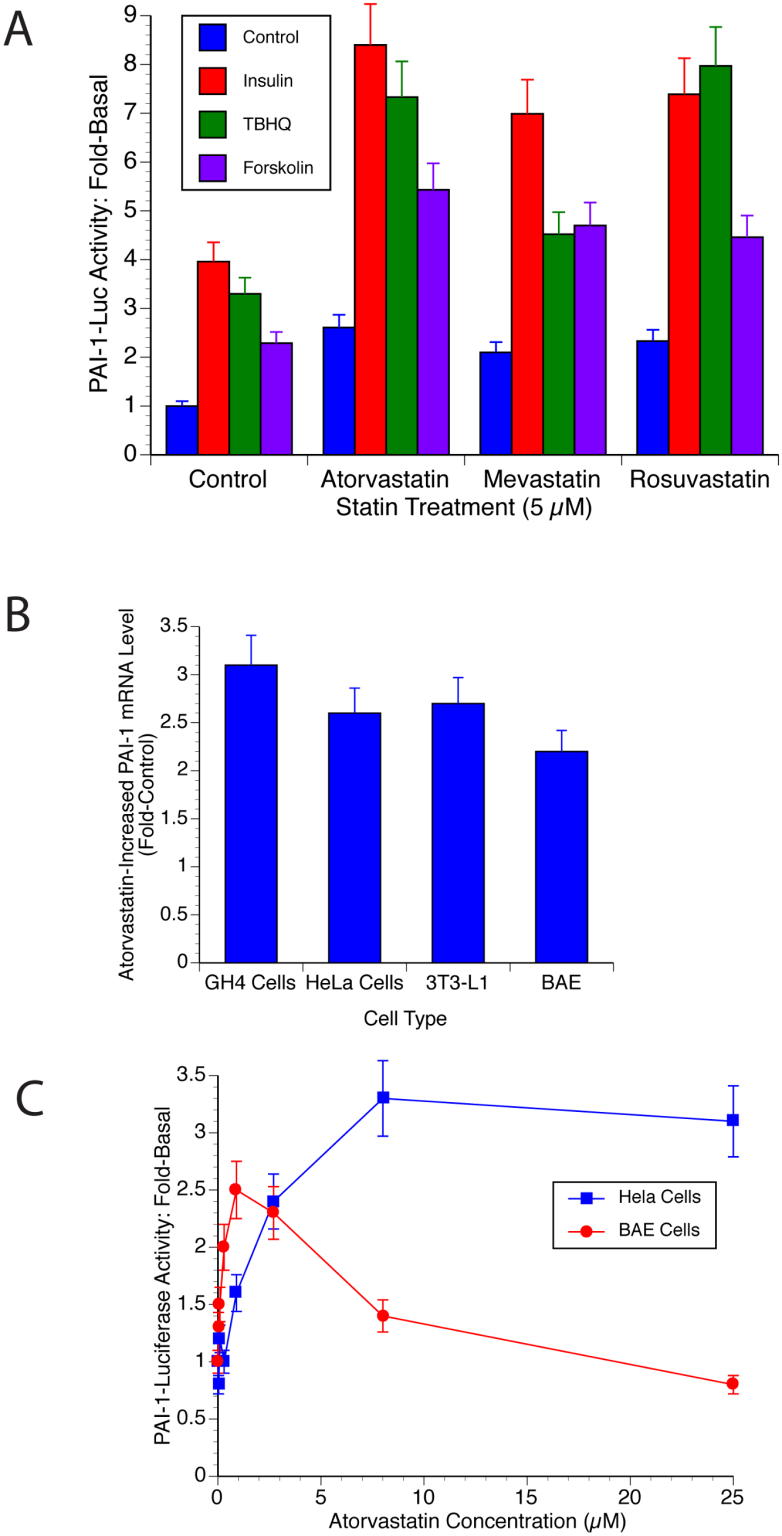
Statins Activate the PAI-1 Promoter. A. Statins increase PAI-1-Luc expression. GH4 cells were electroporated with PAI-1-Luc and RSV-β-Gal as described in Materials and Methods. The cells were incubated for 24 hours with the indicated concentration of atorvastatin, mevastatin or rosuvastatin or with DMSO as a control. Four hours following the addition of the statin, insulin (1 μg.mL), tBHQ (1 μM) or forskolin (1 μM) were added as indicated. The Luc activity was assayed and normalized to the level of β-Gal expression. All values were then normalized to the untreated control (basal). B. Statins increase PAI-1 mRNA. GH4, HeLa, 3T3-L1 and BAE cells were incubated for 24 h with 10 μM atorvastatin or with DMSO (vehicle). RNA was prepared and reverse transcribed as described in Materials and Methods. The cDNA was then analyzed using qPCR. C. The effect of statin is dose-dependent. HeLa and BAE cells were incubated with the concentration of atorvastatin shown for 24 h. The cells were harvested and PAI-1 mRNA levels were determined as described in Materials and Methods.

We investigated if statins affected the PAI-1 promoter because atorvastatin decreased PAI-1 levels in adipose tissue of atherosclerotic rabbits [25]. It was also previously reported that certain nuclear receptors affected PAI-1 transcription, and statins are known to act as ligands for some nuclear receptors [19]. Atorvastatin (Lipitor), mevastatin (Compactin) and rosuvastatin (Crestor) increased basal PAI-1-Luc activity 2.5-3-fold in cultured cells (Fig 1A). GH4 cells, rat pituitary tumor cells, are shown but similar results are also seen in HeLa and T47D cells (not shown). Statins also doubled the effects of inducers of PAI-1 transcription. Insulin normally increased PAI-1-Luc activity 4- to 5-fold in GH4 cells. This effect was doubled by atorvastatin treatment to 9- to 10-fold (Fig 1A). Similarly, inducers of oxidative stress such as tert-butylhydroquinone (tBHQ) increased PAI-1-Luc 3-fold and this too doubled resulting in a 7-fold increase with statin treatment (Fig 1A). The effect of cAMP (forskolin) also increased from 2-fold to 6-fold. These are highly significant increases (p<0.005).

These studies were done using reporter assays as a surrogate for the endogenous promoter. In order to establish whether the endogenous promoter behaved in a similar fashion, cells were incubated alone or with atorvastatin for 20 h and PAI-1 mRNA levels were assayed by reverse transcription (RT) followed by qPCR. As seen in Fig 1B, quantitative RT-qPCR of PAI-1 mRNA in GH4, HeLa, 3T3-L1 and bovine aortic endothelial (BAE) cells following statin treatment showed a similar 2- to 3-fold increase in PAI-1 mRNA that supports the data obtained using reporter assays.

Finally, Fig 1C shows the dose dependency of statin-increased PAI-1 mRNA in HeLa and BAE cells. Transformed cells seem to require relatively high doses of statins in the low μM range to produce their effects (Fig 1C). BAE cells are primary cells and were quite sensitive and responded maximally to statins at doses normally achieved during statin therapy. The drop-off in statin effect at high doses was most likely due to toxic effects since these cells are very sensitive to xenobiotics and are easily killed by drugs/inhibitors.

### Statin activation of PAI-1 gene transcription is not mediated by inhibition of HMG-CoA reductase

**Fig 2:**
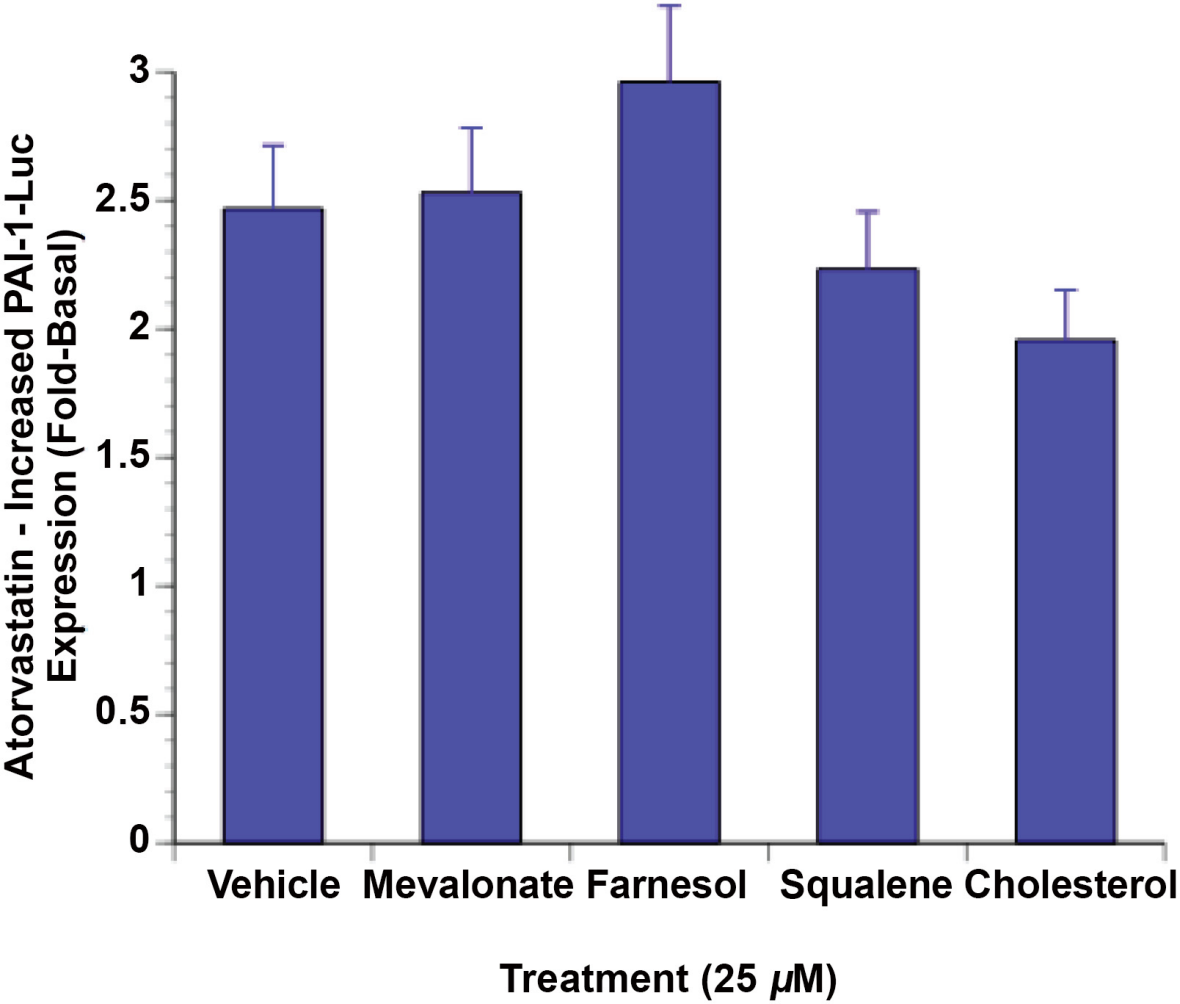
Statin activation of PAI-1 gene transcription is not mediated by inhibition of HMG-CoA reductase. GH4 cells were electroporated with PAI-1-Luc and RSV-β-Gal as described in Materials and Methods. The intermediates of cholesterol metabolism shown in the figure were added at 25 μM the day following electroporation. They were then treated with 25 μM atorvastatin for 20 h. Luciferase activity was then determined and normalized to β-Gal and to the untreated control.

One way that statins could affect gene transcription is by reducing the concentration of intermediates of cholesterol biosynthesis since some of these are ligands for nuclear receptors. This was tested using a number of the intermediates of cholesterol synthesis that are downstream of HMG-CoA reductase. Fig 2 shows that the effect of statins is unchanged when the medium was supplemented with mevalonate, squalene, farnesol, or cholesterol. Thus, it was unlikely that the effect of statins was due to their inhibition of HMG-CoA reductase.

### Statins act through a DR + 3 at -269/-255

Statins were found to affect transcription through nuclear receptors. Statins inhibit the production of oxysterols that act as ligands for the liver X receptors (LXR) [20,21]. Simvastatin was also shown to reduce the expression of farnesoid X receptor (FXR) [26]. PXR and constitutive androstane receptor (CAR) were also transcriptionally activated by statins [24]. In this study, the authors also demonstrated *in silico* compatibility of pravastatin with PXR. Das *et al*. found that statins inhibit a subset of nuclear receptors through inhibition of farnesyl pyrophosphate accumulation [19] and *in silico* modeling showed that farnesyl pyrophosphate docked well to the ligand binding pocket of TR and other classical steroid receptors. PXR [24,27,28] and the constitutive androstane receptor (CAR) [24] were also transcriptionally activated by statins. In this later study, the authors also demonstrated *in silico* compatibility of pravastatin with PXR.

**Fig 3:**
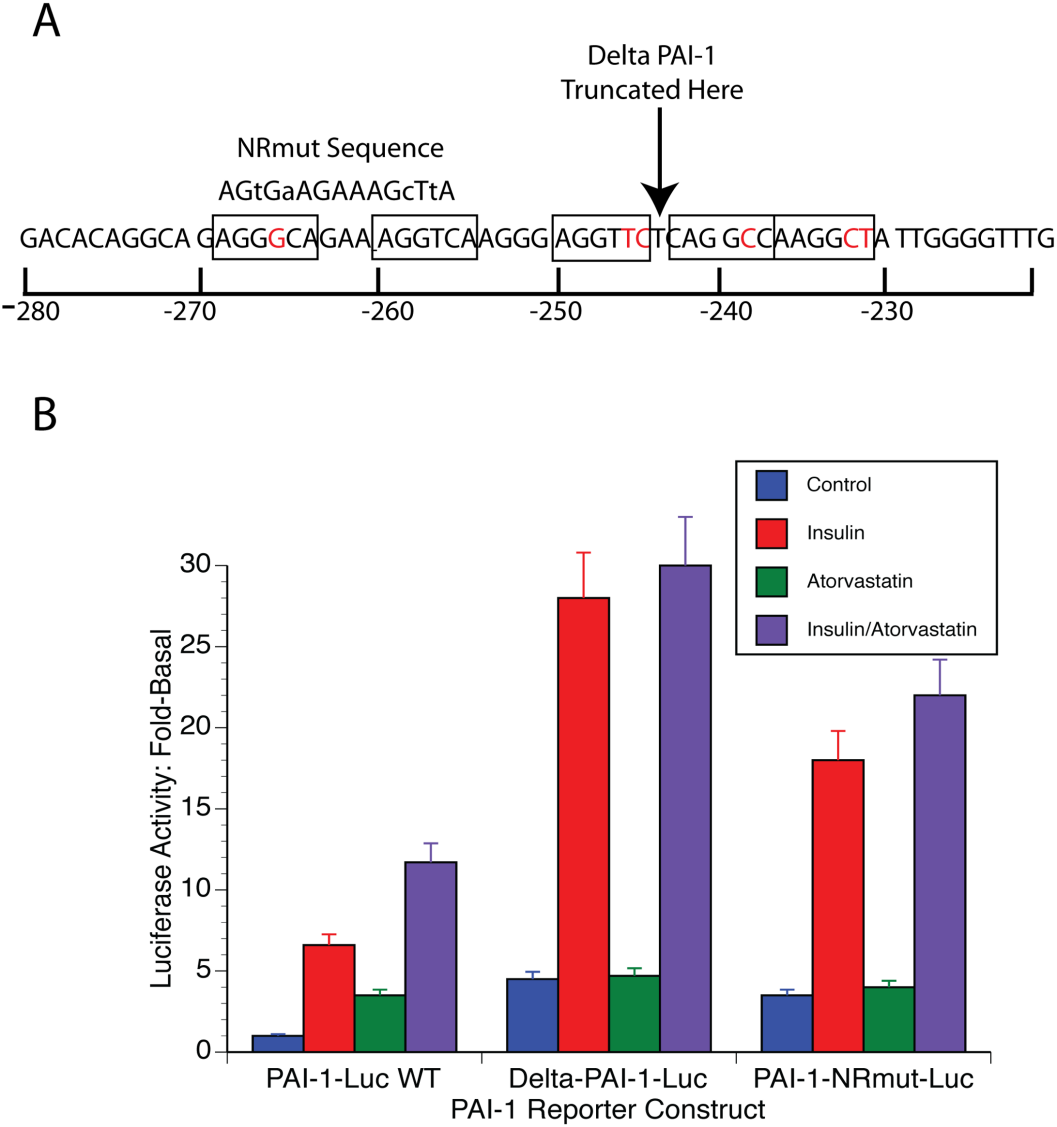
DNA sequence responsive to statins. A. An analysis of the PAI-1 promoter between -280/-220 showed 5 potential nuclear receptor response element half-sites of AGGTCA (boxed). Deviations from the consensus are indicated in red. The site of truncation of the PAI-1 promoter in the plasmid ΔPAI-1-Luc is shown by an arrow. The sequence of the mutant PAI-1 promoter of the PAI-1-NRmut-Luc reporter is shown above the wild type sequence. The lower case letters in this sequence are the mutated bases that were introduced. B. The atorvastatin response of wt-, ΔPAI-1-, and PAI-1-NRmut-Luc is shown in the left, center and right of this graph. The activity of the wtPAI-1-Luc reporter with only vehicle has been set to 1. Atorvastatin, 10 μM and/or insulin, 1 μg/ml, were added to the indicated cultures for 18 hrs.

The nuclear receptor family of sequence-specific transcription factors binds to DNA containing the sequence AGGTCA either as direct repeats with variable length of intervening nucleotides or as inverted repeats with spacing or as a palindrome [14]. Fig 3A shows that a number of potential nuclear receptor half-sites are present in the PAI-1 promoter (underlined) between -280/-221 with the most likely nuclear receptor response element being between -269/-255 (AGGGCANNNAGGTCA). This element was deleted in the reporter plasmid ΔPAI-1-Luc (Fig 3A, Materials and Methods). This reporter had a 2.5-fold higher level of basal expression than the wild type promoter (Fig 2B) and statins did not increase PAI-1-Luc expression using this reporter (Fig 2B). Furthermore, statins had no effect on stimulated expression with this reporter. Thus insulin treatment increased luciferase activity 6-fold over the new higher, basal levels, but the insulin plus statin treatment is 5.5-fold controls which was not significantly different. Likewise, tBHQ increased the activity of this promoter by 4-fold while tBHQ plus atorvastatin produced a similar 3.5-fold increase.

This suggested that it was likely that the effect of statins was mediated through the DR + 3 at -269/-255. To confirm this, we made a PAI-1-Luc with specific mutations of this sequence in the context of the -800 PAI-1 promoter construct (Fig 3A). This reporter also had a 3-fold higher basal activity than the wild type reporter and its activity was not increased by statins (Fig 3B). Again, stimulated expression was also unaffected by statins. Insulin-increased activity was 7-fold alone and 7.5-fold with statins. tBHQ-increased PAI-1-Luc expression was 4-fold alone and 4.3-fold with statins. This demonstrated the importance of this response element for promoter activation by statins. Moreover, it suggested that transcription factor/s binding this element might repress the activity of the promoter in the unbound state.

### Statins can directly activate PXR

**Fig 4:**
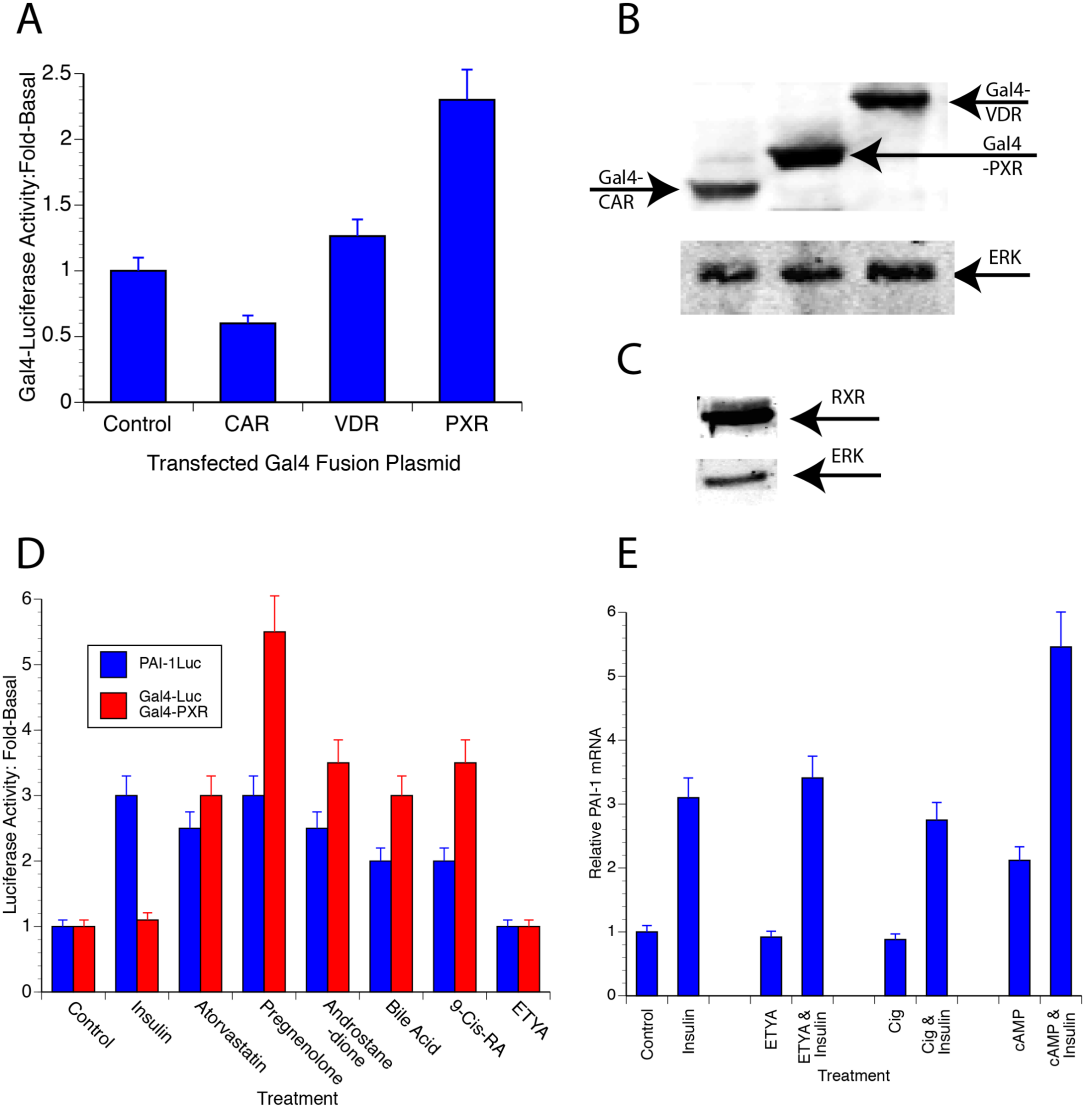
A PXR/RXR heterodimer may mediate the effects of statins. A. Fusion proteins consisting of the Gal4 DNA-binding domain and the ligand-binding domain of a number of heterodimerizing nuclear receptors (indicated in the Fig) PXR, CAR or VDR were expressed in GH4 cells along with a Luc reporter that contained 5 repeats of the Gal4 binding element and an expression vector for RXRα. Control transfections contained only a plasmid expressing the Gal4 DNA-binding domain, with the Gal4-Luc reporter and RXRα. Atorvastatin was added for 16–18 h and Luc activity was determined and normalized to β-Gal as described. B. All of the Gal4 fusion proteins were expressed as determined by western blotting using an antibody to the Gal4 DBD (upper). Anti-Erk ½ was used as a loading control (lower). C. RXRα was expressed as determined using an antibody to RXRα (upper). Anti Erk 1/2 was used as a loading control (lower). D. The ligand specificity of Gal4-PXR/Gal4-Luc is compared with that of PAI-1 promoter. GH4 cells were electroporated with PAI-1-Luc and RSV-β-Gal or with Gal4-PXR, Gal4-Luc and RSV-β-Gal as described in Materials and Methods. The cultures were treated with 10 μM of the indicated potential agonist after 24 h. The cultures were harvested 20 h later and Luc activity was determined and normalized as described (Materials and Methods). E. PAI-1 mRNA levels in GH4 cells treated with insulin, cAMP, ETYA and/or ciglitazone. GH4 cells were treated with 1 μg insulin, 0.5 mM 8-CPT-cAMP, 10 μM ETYA or 10 μM ciglitazone for 24 h as indicated in the figure. RNA was prepared and reverse transcribed as described in Materials and Methods. The cDNA was then analyzed using qPCR.

That statins acted through a DR + 3 suggested that statins might activate PXR, CAR or VDR since they primarily bind to a DR + 3 response element [14]. Gal4-nuclear receptor chimeras provided a way to determine which of the nuclear receptors were activated by statins. These experiments used chimeric proteins consisting of the Gal4 DNA-binding domain at the N-terminus and the LBD of a nuclear receptor at the C-terminus. The transactivating potential of the chimeric protein was measured using a 5X-Gal4-Luc reporter plasmid. This system eliminated the need to compare results from multiple reporters driven by different response elements that would give varying basal and stimulated responses. Fig 4 shows the effect of atorvastatin on these chimeric proteins. All of these constructs were expressed in GH4 cells (Fig 4B) along with RXRα (Fig 4C) that is necessary for maximal activity of these constructs. Gal4-PXR was unique among these since atorvastatin increased its effect on Luc expression 2.5-fold. Atorvastatin had no significant effect on VDR and CAR. This showed that statins directly activated PXR and suggested that statin-activation of PXR led to increased PAI-1 transcription.

If statins acted through PXR to increase PAI-1 gene transcription, then ligands that activate Gal4-PXR also should also activate the PAI-1 promoter through endogenous PXR (Fig 4D). Atorvastatin, pregnenolone, androstanedione and bile acid increase Gal4-Luc activity between 3- to 5-fold in Gal4-PXR transfected cells while they activate PAI-1-Luc between 3- to 4-fold. ETYA, which is an agonist for PPARs, do not activate Gal4-PXR or PAI-1. Only insulin that activates PAI-1 through a Forkhead response element at -52 increases PAI-1-Luc while not having any effect on Gal4-PXR

Since ETYA did not activate PAI-1-luc or Gal4-Luc, we hypothesized that it would not increase endogenous PAI-1 mRNA expression. When GH4 cells were treated with ETYA or ciglitazone that activate PPARα or PPARγ respectively, they did not activate PAI-1 mRNA (Fig 4E) while insulin and cAMP increased PAI-1 mRNA and the effect of cAMP was additive with that of insulin (Fig 4E). This was consistent with the insulin activation of PAI-1 through FoxO3a [10] and cAMP activation through AP-1 [29]. ETYA and ciglitazone do not activate PAI-1 mRNA either without or with insulin since PPARs do not activate the response element. This is in contrast to atorvastatin that activates PAI-1-luc (Fig 1A and Fig 4D), Gal4-PXR mediated Gal4-Luc expression (Fig 4D) and PAI-1 mRNA (Fig 1B) likely through PXR.

### *In Silico* Docking of Statins to PXR

**Fig 5:**
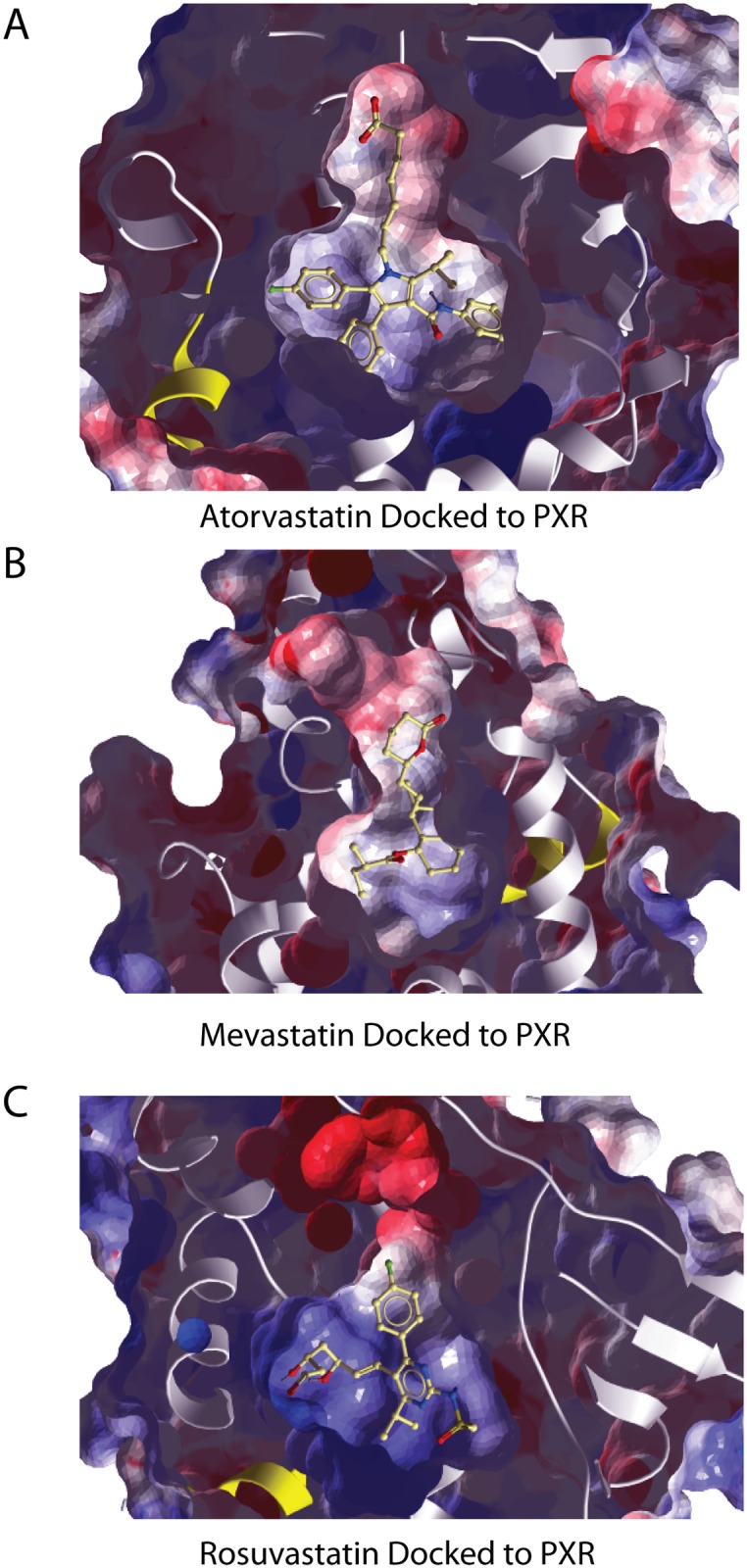
*In Silico* statin docking to PXR. A panel of statins was docked to crystal structures of PXR in several conformations including apo forms and ligand-bound forms as described in Materials and Methods. All of the statins tested docked with PXR with energies and energy gaps (see Materials and Methods for the definition of energy gap) that were indicative of specific interactions between PXR and the statins tested (Table 1). The figure shows atorvastatin (A), mevastatin (B), and rosuvastatin (C) docked within the receptor-binding pocket of an apo PXR structure (4J5W). Statins are ball and stick representations while PXR is shown in a space filling representation with alpha helices in white. Red and blue shading represent negative and positive environments, respectively. The outer surface of the protein has been made transparent so that the docked statin can be seen within the binding pocket.

A previous study [24] used computational molecular docking to model the interaction of pravastatin and lovastatin with an agonist- induced PXR 3D structural conformation (PDB: 1SKX). Atorvastatin, mevastatin and rosuvastatin should also dock well with PXR if they are PXR agonists. We used small molecule docking (Materials and Methods) to evaluate the 3D structural compatibility of atorvastatin, mevastatin and rosuvastatin to the ligand pocket of an apo form of PXR (pdb: 4J5W). They all bind with favorable energies (Table 1) suggesting that they are ligands for PXR that are comparable to those of documented PXR ligands such as rifampicin. The hydrophobic aromatic groups of the statins are seen to be in close proximity to the segment denoted helix 12 (Fig 5), known to be important for coactivator binding [30]. However, crystallographic structures of agonist-bound, antagonist-bound and apo forms of PXR do not show the characteristic shift in helix 12 that has been shown in other nuclear receptors. Thus, the PXR activation mechanism may have more to do some other ligand-induced change in the LBD, such as stabilization, than with any positional shift in helix 12.

**Table 1:** ICM Docking Scores for Common Statins.

Ligand	Docking Score	Energy Gap	PDB ID
Atorvastatin	-36.58	6.81	2o9i
Lovastatin	-31.86	4.02	4j5x
Mevastatin	-28.16	6.68	2o9i
Pravastatin	-28.74	5.96	1ilh
Rifampicin	-34.92	8.64	1m13
Rosuvastatin	-35.78	9.55	4j5x
Simvastatin	-30.29	4.67	4j5x

The ICM docking score and energy gap for a number of common statins is shown. Rifampicin, the canonical PXR ligand, is included as a positive control. The more negative the docking score, the better the fit into the ligand-binding pocket. The energy gap indicates the difference in energy between the lowest and second lowest energy docking. Large energy gaps are indicative of the uniqueness of the low energy docking conformation. The fourth column lists the PDB IDs corresponding with the 3D structures used to compute the docking scores and energy gaps in the table.

### PAI-1-Luc Expression is PXR dependent

**Fig 6:**
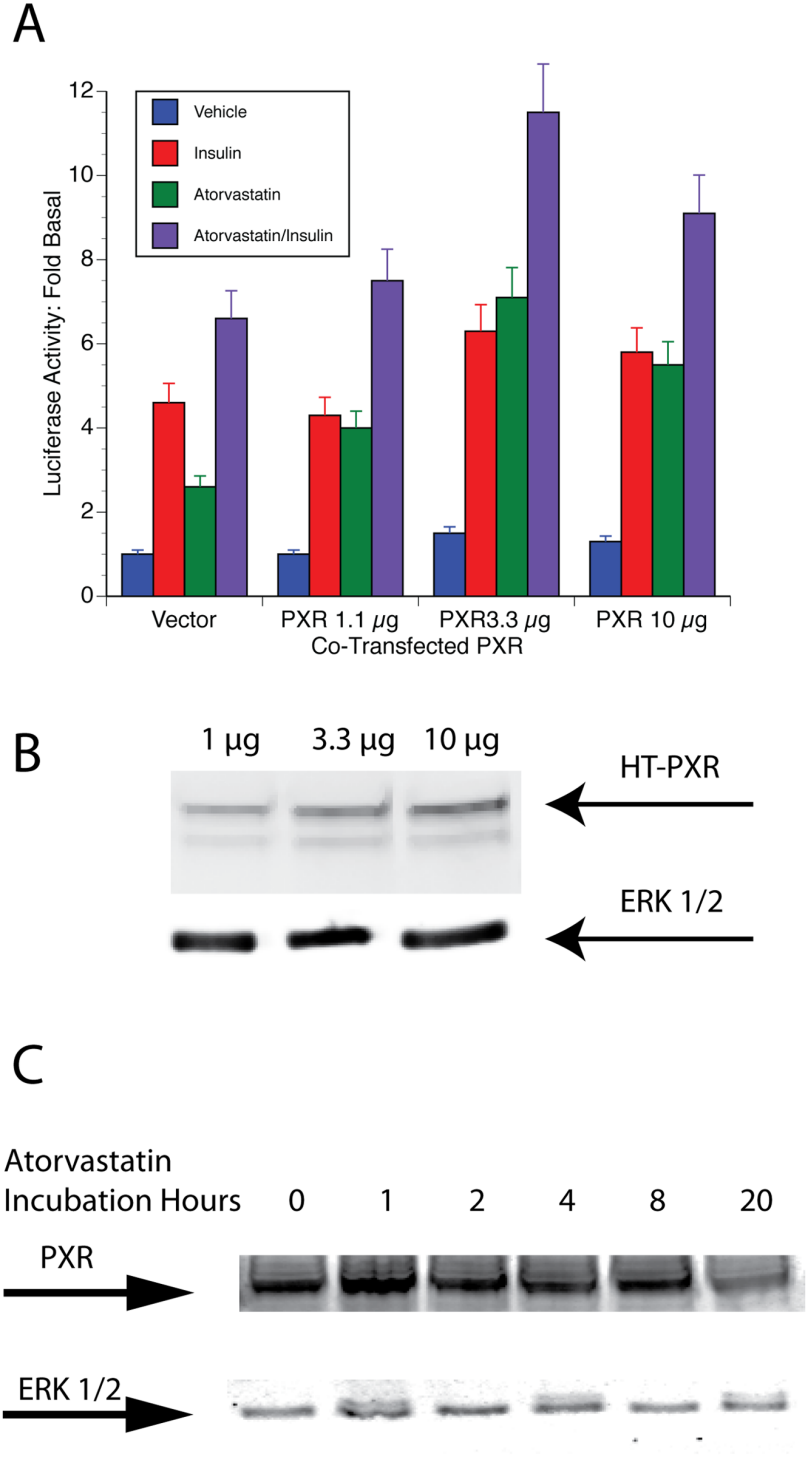
Expression of PXR affects PAI-1-Luc activity. A. An expression vector for Halo-tagged PXR at concentrations indicated in the figure was transfected into GH4 cells along with the PAI-1-Luc reporter plasmid. After 24 h, 10 μM atorvastatin (green bars), 1 μg/mL insulin (red bars) or both (purple bars) were added to the cultures. The cells were harvested after an additional 20 h and analyzed for PaI-1-Luc expression as described (Materials and Methods). The blue bars indicate control cells. B. Expression of Halo-tagged PXR. Nuclear extract from cells treated as in A was prepared and analyzed for expression of Hallo-tagged-PXR as described in Materials and Methods. Nuclear Erk ½ was used as a loading control. C. Atorvastatin does not affect the expression of PXR—GH4 cells were incubated with10 μM atorvastatin for the times indicated. The cells were harvested and a nuclear extract was resolved on SDS-PAGE and analyzed via western blot with anti-PXR and anti-ERK 1/2 as a loading control (Materials and Methods).

The DR + 3, the Gal4 studies and the metabolite experiments suggested that PXR might be critical to PAI-1 promoter activity. If PXR acts at the nuclear-receptor response element at -269/-255, then PXR overexpression might be expected to increase atorvastatin responsiveness of the promoter by increasing occupancy of the site. GH4 cells that were transfected with increasing amounts of an expression vector for PXR had increased levels of PXR (Fig 6B). Although cotransfection with 1 μg of HT-PXR had no significant effect on PAI-1-Luc expression, basal PAI-1-Luc expression increased 1.5-fold with 3.3 μg and 10 μg of HT-PXR expression vector. Atorvastatin increased the expression of PAI-1-Luc 2-fold from 3- to 6-fold in the presence of added PXR. Furthermore, additive increases in stimulated expression were also seen. Insulin stimulation increased from 5-fold to 7-fold and atorvastatin plus insulin increased from 8-fold to 12-fold.

A previous study had demonstrated that statins could increase FXR levels. This suggested that statins might affect PXR levels in these cells. Cells were treated for various times with 5 μM atorvastatin, and PXR levels in cell lysates were analyzed by western blotting. PXR expression was not changed by atorvastatin treatment during this experiment (Fig 6C).

### PXR and RXR associated with the PAI-1 promoter

**Fig 7:**
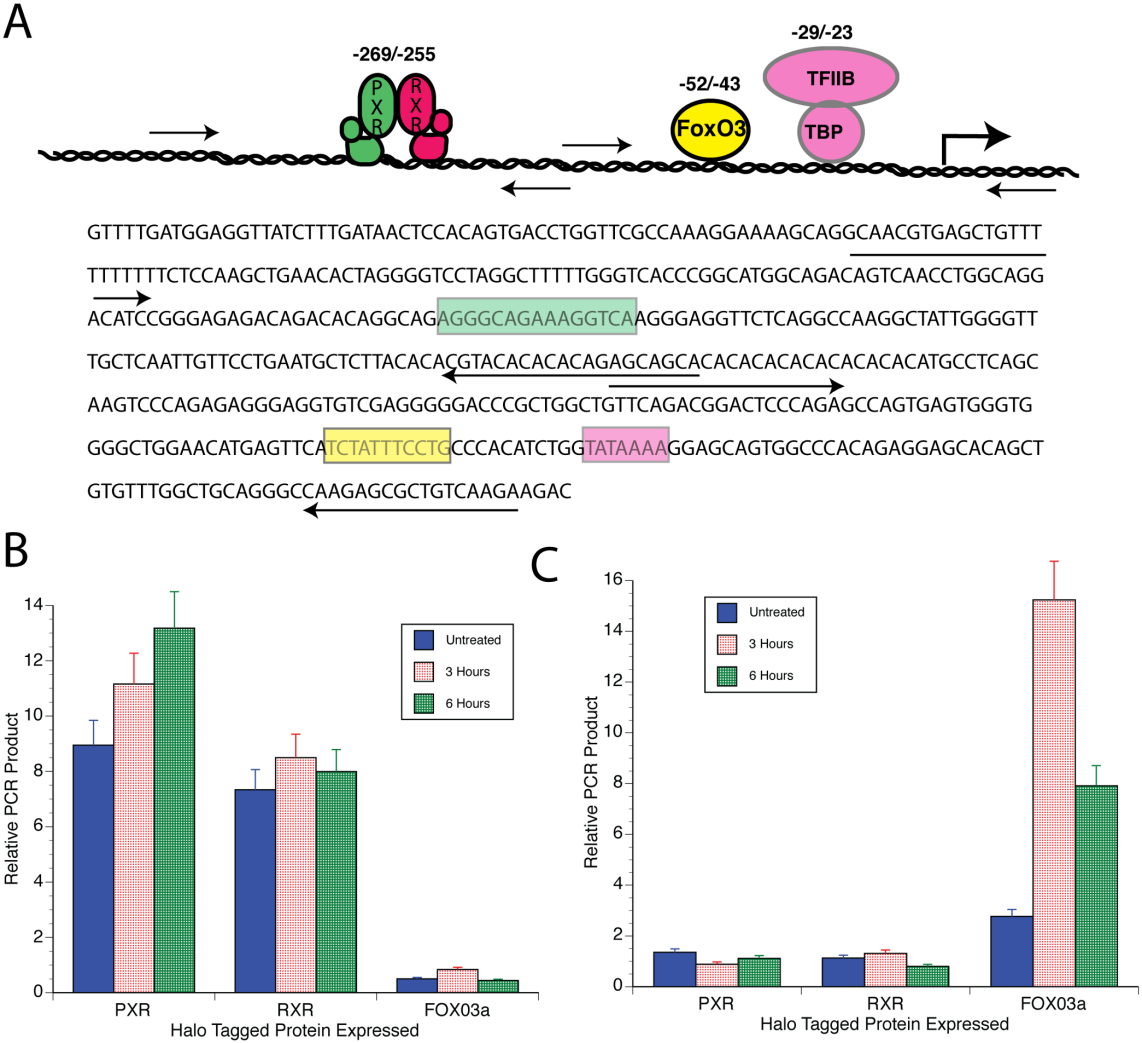
Halo-ChIP analysis of the PAI-1 promoter. Halo-tagged fusion constructs of PXR, RXRα, and FoxO3a were made as described in Materials and Methods and transfected into HeLa cells. Atorvastatin (PXR/RXRα) or insulin (FoxO3a) were added for the times indicated and chromatin was cross-linked using 1% formaldehyde for 10 m. The chromatin was then sheared and the HaloTag containing protein-DNA complexes were isolated using HaloLink resin (Promega). The DNA was then de cross linked by heating at 60°C overnight. The isolated DNA was then passed through a column (Fermentas) to remove any contaminating proteins and qPCR was performed as described in Materials and Methods. A. Schematic of the PAI-1 promoter with hypothetical binding sites and primers used in pPCR analysis (A, top) and the sequence of the PAI-1 promoter in this region (A, bottom). The green colored box is the putative nuclear receptor response element. The yellow box is the Fox binding element. The rose colored box is the TATA element. Arrows under the sequence show the direction and sequence of PCR primers. B. Results with primers amplifying the distal promoter. C. Results using primers to the proximal promoter.

ChIP experiments were performed to determine if PXR and RXR could be found associated with the PAI-1 promoter in the region that we determined to be statin sensitive. Fig 7A shows various regions of the PAI-1 proximal promoter and the PCR primers that we used to analyze them. Fig 7B and 7C show the results of our analysis. GH4 cells were transfected with halo-tagged PXR, halo-tagged RXR or halo-tagged FoxO3a. FoxO3a was used as a control since we previously demonstrated its association with the PAI-1 promoter at a position downstream of the putative nuclear receptor element at -265/-255 [10]. Atorvastatin was added to the cultures 24 h after transfection for the times indicated. Then the chromatin was crosslinked with 1% formaldehyde for 10 min. at room temperature. The chromatin was isolated and precipitated as described in Materials and Methods. Both PXR and RXR bind to the area of the PAI-1 promoter containing the -269/-255 nuclear receptor response element (Fig 7B) while FoxO3a, which was previously shown to bind to a more proximal region of the PAI-1 promoter does not [10]. Conversely, FoxO3a binds to the proximal PAI-1 promoter (Fig 7C), but RXR and PXR do not. Statins do not significantly affect the amount of PXR or RXR bound to the promoter (Fig 7B).

## Discussion

These studies identify an important and previously poorly defined regulatory element in the PAI-1 promoter that is responsive to statins. This element is a classical nuclear receptor binding site with a DR + 3 at -269/-256 (Fig 3). The binding of PXR to this element (Fig 7), the activation of Gal4-PXR by statins (Fig 4), the ligand specificity of the response (Fig 5) and the *in silico* docking of statins to PXR (Fig 5) make it likely that statins activate PXR bound at -269/-255. The observation that elimination of this element results in higher levels of PAI-1 expression (Fig 3) suggests that the transcription factor(s) that bind to this element inhibit PAI-1 expression. This is consistent with the recruitment of corepressors to the promoter by some unliganded nuclear receptors [14]. A number of corepressors have been identified including the nuclear receptor corepressor (NCoR) and its homolog, the silencing mediator of retinoid and thyroid receptors (SMRT). Alternately, the binding of the unliganded receptor to this element could affect other transcription factors or the conformation of the chromatin in its proximity.

Two groups had previously identified part of this element, -261/-256, but had missed the upstream half-site [31,32]. The first group attributed the promoter activation to Nur77/NAK-1 (NURR-1) based on electrophoretic mobility shift assays and similar patterns of stimulation of NUR77 and PAI-1 in response to TNFα [31]. However, their EMSA studies used a -270/-250 bp probe that included both half-sites and thus could have bound PXR and/or other nuclear receptors. Since they used different cells than used in this study, it is possible that NUR77 mediated a response in those cells at this element.

The second group identified an upstream hemisite at -418/-409 and a downstream hemisite at -265/256 [32]. Deletion experiments do not support the upstream site, at least in the cell lines used in these studies. Interestingly, however, they attributed activity at this site to Rev-erbA alpha that inhibited PAI-1 transcription through these elements. Therefore, it might be possible that in the absence of statins, Rev-erbA alpha inhibits transcription at this site only to be replaced by an RXR/PXR heterodimer following statin binding. We have no evidence for this and the data are most consistent with PXR/RXR heterodimer mediating both inhibition and stimulation at this site in the cells that we studied.

Since first proposed in 1991 [15], the “3, 4, 5” rule of nuclear receptor binding that we used here to help predict the element responsive to statins has been an important model for locating nuclear receptor promoter elements. Developed using reporter assays in tissue culture and in vitro DNA-binding models such as electrophoretic mobility shift assays, it has allowed the prediction of responsive promoters and mutant sites. Recently, the purified PXR/RXR heterotetramer was used to predict binding affinity of various DNA sequences in vitro [33]. This study utilized the PXR response element of the CYP3A4 that is a DR + 3. Recently, this model has been extended with somewhat surprising results [34]. When ChIP/Seq was used to examine PXR binding in mouse liver, different half-site spacings were found. The optimal PXR binding site found in this study was a DR (+ 4) or DR(5n + 4). Since PXR binding sites were correlated with marks of active chromatin (histone H3K4 dimethyl) but not with inactive chromatin (histone H3K27 trimethyl), it could be chromatin configuration-dependent. Why liver chromatin should be different than chromatin in tissue culture cells is unclear and awaits clarification.

Our studies suggest a direct effect of statins to activate PAI-1 transcription through PXR since Gal4-PXR was the only nuclear receptor tested that was activated by statins (Fig 4A). The other DR + 3 binding nuclear receptors, VDR and CAR, were neutral in this assay. Further, the ligand responsiveness of Gal4-PXR was close that of PAI-1-Luc (Fig 4). Finally, PXR binds to the PAI-1 promoter in ChIP experiments (Fig 7).

Statins were shown to act through nuclear receptors to increase transcription of many genes. The best studied of these is the promoter for cytochrome p450 3A (CYP3A), which was responsive to most statins through PXR, CAR, and FXR, with PXR responding most vigorously and to the largest number of statins [24]. Docking of pravastatin to the PXR ligand-binding domain showed a good structural fit despite pravastatin being among the weaker inducers of CYP3A. Our *in silico* analysis demonstrated that all of the statins examined docked with PXR sufficiently well to be predicted as ligands. The aromatic residues of the statins appear to make close contact with helix 12 of PXR. This might cause an alteration in PXR structure that would lead to coactivator binding or these ligands may be associated with activation by some other mechanism.

It seems likely that the docked complex of statins with PXR could be used to design better statins using structure-based design. Since the structure of HMG-CoA reductase is known, statins could be designed to interact with HMG-CoA reductase, but not to fit the ligand-binding pocket of PXR. Thus, an improved statin with lower side effects can be theorized. Alternatively, a number of approaches have been used to design antagonists for PXR that could be used in association with statins to block statin effects on PXR [35–37]

Complicating this analysis, products of HMG-CoA reductase have many other effects on nuclear receptors and transcription. LXRα regulated by geranylgeranol that inhibited the constitutive activity of LXRα [23]. A subsequent study showed that geranylgeranyl pyrophosphate antagonized the interaction between LXRα or LXRβ and SRC-1, a nuclear receptor co-activator [38]. *In silico* modeling showed that thyroid hormone receptor and a number of classical steroid receptors could bind farnesyl pyrophosphate [19]. Subsequent analysis showed that these receptors were activated by farnesyl pyrophosphate while PPARs, LXR and FXR were not activated. However, neither mevalonate nor squalene affected the statin-mediated increases that we observed in PAI-1 gene transcription. This argues strongly against an effect of cholesterol metabolites on the response.

These results point to a direct activation of PXR by statins, but do not rule out other effects of statins acting through HMG-CoA reductase pathway-dependent compounds. Studies have found that decreased geranylgeranyl pyrophosphate inhibits the Rho-GTPase pathway that normally modulates LXR [21]. Since we previously showed that Rho could activate transcription, it seems more likely that inhibition of Rho signaling would have a negative effect on PAI-1. Others have shown that lovastatin can activate Ras through an effect on phospholipase D2 [20]. This might contribute to the activation of PAI-1, but the MEK inhibitor U0126 does not affect PAI-1 transcription (unpublished data) and thus it is not likely that this is an issue in the present study. Statins were also shown to decrease FXR levels in Syrian hamsters [39]. The level of PXR was unaffected in our cell culture systems by the statins that were used. However, this could be a factor with other statins or when statins are assessed in vivo.

The increased basal PAI-1-Luc expression that results when the DR + 3 is mutated suggests that the PXR response element has a repressive effect in the absence of ligands. It will be important to define this process in more detail. This will include studies to determine coactivator/corepressor binding as well as chromatin modification at this site.

The statin activation of PXR and the resulting activation of PAI-1 promoter has important theoretical and clinical implications. Hypothetically, the effects of statins on PXR at the PAI-1 promoter suggest that unliganded PXR/RXR can in some instances be inhibitory. This is a new observation about PXR as far as we know and requires more detailed follow-up. It is important to determine the recruitment of corepressors and how they are overcome by physiological stimuli. Clinically, statin activation of an acute-phase protein could account for some of the adverse reactions that are reported with statins and might reduce the overall potential of these drugs to mitigate the deleterious effects of elevated cholesterol. This may account for the failure of statins to achieve meaningful primary protection from heart attacks [39–41].

Presently, we know of no studies in humans that have examined the effect of statin therapy on PAI-1 levels. Elevation of PAI-1 might not make much difference for relatively healthy individuals, but could be critical in such vulnerable populations as diabetics or those with other chronic diseases since PAI-1 is associated with the sequelae of diabetes such as heart and kidney disease [7]. Additionally, PXR activates many drug metabolizing enzymes including CYP3 family members, UDP-glucuronosyltransferases, glutathione S-transferases and transporters such as the multidrug resistance transporters [34]. Thus, statins could also affect other therapies important for the treatment of the chronically ill. The potential to use the docking of new drugs to PXR as a way to design drugs without these side effects or to inhibit negative effects of statins on PXR with PXR inhibitors [36] could be transformative.

## Materials and Methods

### Materials

Restriction enzymes were obtained from Fermentas or New England Biolabs and were used as recommended. Oligonucleotides were from Operon and reagents for PCR were obtained from Fermentas. Dulbecco's modified Eagle's medium containing 4.5 g/L glucose and iron-supplemented calf serum were obtained from Hyclone Laboratories. All other reagents were of the highest purity available and were obtained from Sigma, Bio-Rad, Fisher, or Cal Biochem.

### Cell Culture

GH4 pituitary tumor cells, 3T3-L1 and HeLa cells were obtained from the American Type Culture Collection (Manassas, VA) and maintained in Dulbecco’s modified Eagle’s medium with 10% iron-supplemented calf serum. Bovine aortic endothelial cells (BAE) were the gift of Dr. M. Yorick (U. of Iowa, Iowa City, IA) and were maintained in Dulbecco’s MEM with 10% iron-supplemented calf serum. All experiments were done in medium containing charcoal-treated serum that was shown to be hormone and growth factor depleted [42].

### Plasmids

The PAI-1 promoter reporter plasmid, p800neo-Luc, was the generous gift of Dr. D. Rifkin (NYU School of Medicine, New York, NY) [43]. We refer to this plasmid as PAI-1-Luc for clarity. A mutant of the putative nuclear receptor response element at -269/-255 in p800neo-Luc was made using the Change-IT site directed mutagenesis system from USB/Affeymetrix. This plasmid is called PAI-1-NRmut-Luc. Finally, 5’ deletion of the PAI-1 promoter (-800/-245 deleted) was made by PCR. This plasmid was called ΔPAI-1-Luc. Human PXR was provided by Dr. S. Kleiwer (U.T. Southwestern, Dallas, TX) and was recloned into pcDNA3.1 using the Eco/Xho sites flanking the PXR cDNA. PXR and RXRα were also cloned into the Halo-vector pFC14K using PCR. PCR primers were designed using Promega’s Flexi Vector design tool. These primers were used to amplify the target DNA, which was then cloned into the Halo-vector using the EcoICRI and PmeI sites, designed into the PCR products. All cloning was verified by sequencing. Gal4-VDR and Gal4-CAR were from Dr. H. Samuels (NYU, New York, NY). Gal4-SXR-LBD was a gift from Dr. B. Forman (City of Hope, San Diego, CA). This plasmid expresses the mouse PXR LBD. The human insulin receptor expression vector, pRT3HIR2, was the gift of Dr. J. Whittaker (Case Western Reserve, Cleveland, OH).

### Antibodies used

Anti-V5 was obtained from Invitrogen (R960). Anti-Gal4 DNA-binding domain (sc-577), anti-Erk1 and anti-Erk2 (sc-93 and sc-154), anti-RXRαsc-553), and anti-PXR (sc-7737) were from Santa Cruz.

### Transient Gene Transfection Facilitated By Electroporation

Electroporation experiments and reporter assays were performed as described [44]. GH4 cells were harvested with an EDTA solution, and 6 x 106 cells were used for each electroporation. Trypan Blue exclusion before electroporation ranged from 95% to 99%. The voltage of the electroporation was 1550 volts. This resulted in Trypan Blue exclusion of 70% to 80% after electroporation. The transformed cells were plated in 96 well dishes (Falcon Plastics) at 1 x 105 cells/well in DMEM with 10% hormone-depleted serum. The cells were allowed to attach and hormones were added for 24 h. The medium was replaced with assay buffer and the plates were frozen at -80°C. Luc assays were performed using reagents and protocols from Promega.

Control of transfection efficiency was performed using an RSV-β-galactosidase. β-galactosidase activity in the cell lysates was determined using Beta Glo (Promega, Madison, WI). Transfection efficiency did not vary significantly among transfections performed at the same time. The relative light units (RLU) of Luc activity was then corrected for minor variations in β-Galactosidase activity by converting the RLU to RLU / β-Galactosidase activity/mg protein. The fold stimulation or inhibition was then determined.

### Preparation of cytoplasmic and nuclear extracts and western immunoblot analysis

GH4 cells were harvested in an hypotonic buffer consisting of 10 mM Hepes, pH 7.5, 10 mM KCl, 0.1 mM EDTA, 0.1 mM EGTA, 1 mM Na_3_VO_4_, 1 mM (NH_4_)_6_Mo_7_O_24_, 10 mM NaF, 10 mM NaP_2_O_7_, 1 mM dithiothreitol, 1 mM AEBSF, and 10 μg/mL aprotinin. They were allowed to swell on ice for 10 min and lysed by addition of NP40 to a final concentration of 0.5%. The nuclei were collected by centrifugation at 1000 x g for 5 min at 4°C and washed once. They were then extracted with a buffer containing 20 mM Hepes, pH 7.5, 0.4 M NaCl, 1 mM EDTA, 1 mM EGTA, 1 mM Na_3_VO_4_, 1 mM (NH_4_)_6_Mo_7_O_24_, 10 mM NaF, 10 mM NaP_2_O_7_, 1 mM dithiothreitol, 1 mM AEBSF, and 10 μg/mL aprotinin. The samples were vortexed for 15 sec at the highest setting and then placed on ice. The process was repeated four times. The nuclear extract was collected after a 10 minute centrifugation at 14,000 x g. Equal amounts of protein were analyzed by SDS-polyacrylamide gel electrophoresis using 10% gels. The proteins were transferred to nitrocellulose membranes (Micron Separations) and immunoblotted using primary antibodies shown in the figure legends.

### Quantitative Reverse Transcriptase-Polymerase Chain Reaction (RT-qPCR) of PAI-1

Total RNA was prepared using Purescript® (Gentra Systems, Minneapolis, MN). The amount of RNA was estimated from the absorbance at 260/280 nm using a NanoDrop Spectrophotometer (Thermo Scientific). Total RNA was reverse transcribed using Superscript III (Invitrogen, Carlsbad, CA). Each reaction used 10 μg of total RNA and oligo dT primers. The cDNA was then amplified using a BioRad iCycler. Reactions for qPCR in 96 well plates typically contained 0.1 ng of cDNA, 200 nM each primer and 10 μl SYBR Green Mix (ABI, Foster City, CA) in 20 μl reaction. Primers for qPCR were designed using Primer3 [45] ("http://frodo.wi.mit.edu/primer3/primer3_code.html"source code available http://primer3.sourceforge.net/) and in all cases spanned an intron/exon boarder. Controls included GAPDH, HPRT and M2B. Primer sequences are available upon request.

### *In silico* docking of statins to PXR

Docking was performed as previously described [46] to assess the mode of interaction of statins with 11 crystallographic 3D structural models of nuclear receptor PXR. Grid potentials, including those for electrostatics, van der Waals, and hydrogen bonding, were generated from the full-atom Protein Data Bank models of the nuclear receptors. A full-atom flexible statin ligand was then docked to these grid potentials using the Biased Probability Monte Carlo procedure and resulting conformations of the ligand within the grid potentials were ranked according to energy score. Calculation for each model took approximately 4 CPU h on a 1.67-gHz PowerPC G4 chip (~900,000 functional calls). The docking procedure results in a predicted energy spectrum of conformations. The difference between the lowest and second lowest energy in the spectrum is the “energy gap”, and is indicative of the strength of the preference of the ligand for the lowest energy conformation over others independent of the overall energy score (Table 1).

### Halo-ChIP—analysis of transcription factor binding

HeLa cells in 100 mm plates were transfected with plasmids that expressed Halo-tagged PXR, RXRαFoxO3a fusion proteins. The plates were re-fed with hormone-depleted serum-containing medium for 20 h and incubated with atorvastatin or insulin for 1 or 3 hours or without additions as controls. The cells were fixed by adding formaldehyde to the medium at a final concentration of 1%. After 10 min at RT, the reaction was quenched with 0.125 M glycine. Nuclei were then prepared and sonicated 15 times for 15 sec each in a Diagenode sonicator. The samples were made 1% Triton X-100 and the size and amount of the fragments was assessed on an agarose gel after decrosslinking. The sonicated chromatin was then incubated with HaloLink resin for 2 h at RT. The Halo-tagged protein is covalently bound to the resin during the incubation. The complexes were then washed 4 times with high salt buffer (700 mM NaCl) and 4 times with H_2_O for 10 min each at RT. The cross-linked chromatin was recovered, purified and analyzed using qPCR (Fig 5B). A Syber Green master mix (ABI) was used with the same primers that were used as for semi-quantitative PCR in a BioRad iCycler. A total of 45 cycles were performed although all samples amplified between 22 and 35 cycles.

Forward and reverse primers were designed using the Primer 3 program (see above). Primers were designed that would amplify a proximal section of the PAI-1 promoter that included the insulin response element at -52/-42 but not the suspected nuclear response element at -269/-255. Another set of primers was designed to a distal region of the promoter that did not contain an IRS but contained the putative nuclear response element at -269/-255. Sequences for the primers are:

PAI.Dist.For, GGGACCATCTAGTTGCAGGA; PAI.Dist.Rev, GGGACTGGTTTCATGGAAGA;

PAI.Prox.For, AGTCCCAGAGAGGGAGGTGT;PAI.Prox.Rev, TCTTCTTGACAGCGCTCTTG

## Supporting Information

S1 Fig Complete western blots of transfected Gal4-Receptor constructs shown in Fig 4 with molecular weight markers and non-specific bands. A. Complete blots from Fig 4B showing the expressed Gal4-CAR, Gal4-PXR, and Gal4-VDR and the Erk control blot. B. Complete blots from Fig 4C showing expression of RXRα and Erk control.
(TIF)

S2 Fig Complete western blots of transfected HT-PXR and endogenous PXR shown in Fig 6 with molecular weight markers and non-specific bands. A. Complete blot of the expression of HT-PXR shown in Fig 6B and the complete Erk control blot. B. Complete blot of endogenous PXR levels in response to atorvastatin shown in Fig 6C and the blot of the Erk control.
(TIF)

## Abbreviations

Luc: luciferase
CAR: constitutive androstane receptor
FXR: farnesoid X receptor
LXR: liver X receptor
PXR: pregnane X receptor
RXR: retinoid X receptor
VDR: vitamin D receptor
Gal: galactosidase
PAI: plasminogen activator inhibitor
Fox: Forkhead box
BAE: bovine aortic endothelial
DBD: DNA-binding domain
LBD: ligand-binding domain
DR: direct repeat
