# Supplementary figures and images for: Statins Increase Plasminogen Activator Inhibitor Type 1 Gene Transcription through a Pregnane X Receptor Regulated Element
Source: PLoS One. 2015 Sep 17;10(9):e0138097. doi: 10.1371/journal.pone.0138097 (PMC4574702; doi:10.1371/journal.pone.0138097)

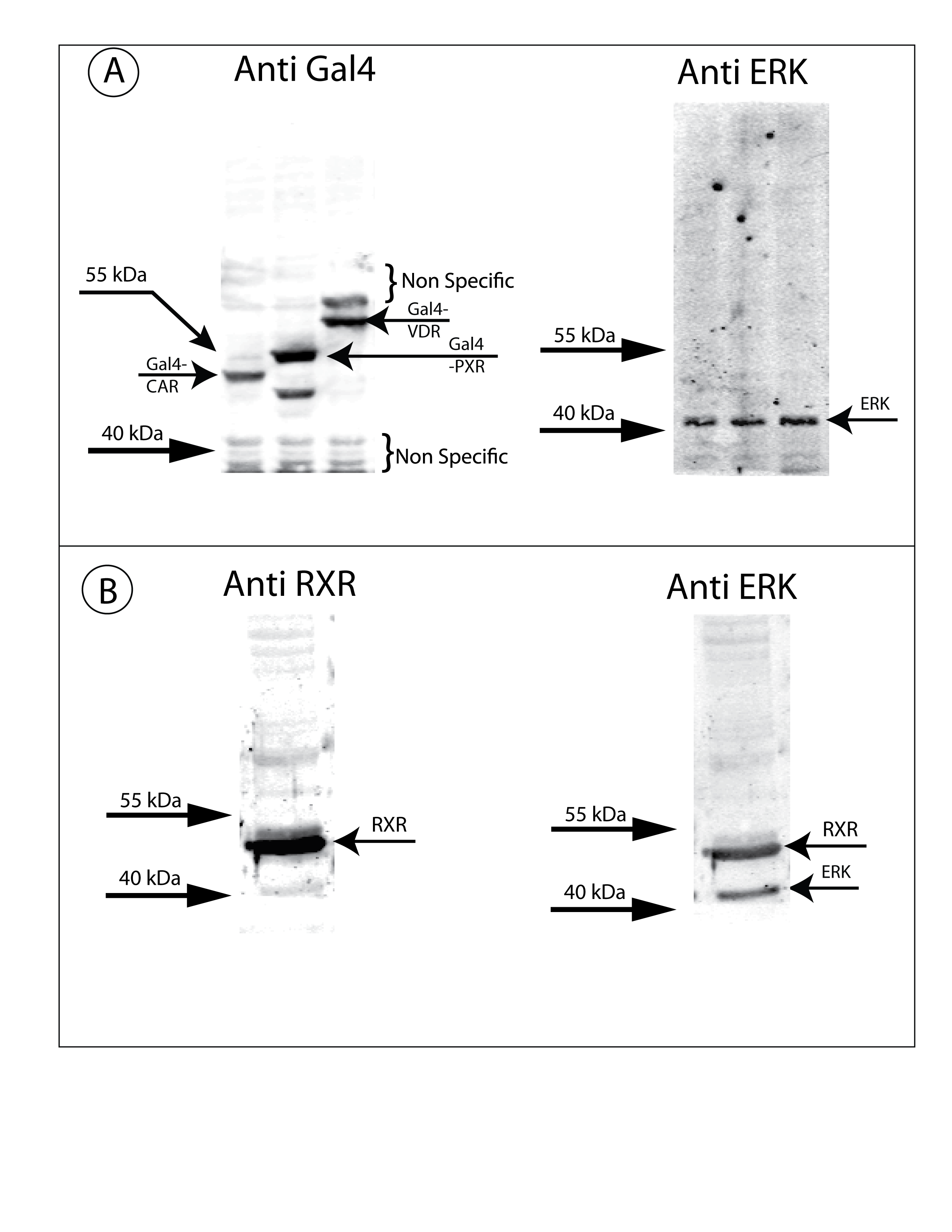

Supplement: S1 Fig — A. Complete blots from Fig 4B showing the expressed Gal4-CAR, Gal4-PXR, and Gal4-VDR and the Erk control blot. B. Complete blots from Fig 4C showing expression of RXRα and Erk control. (TIF) [file pone.0138097.s001.tif]

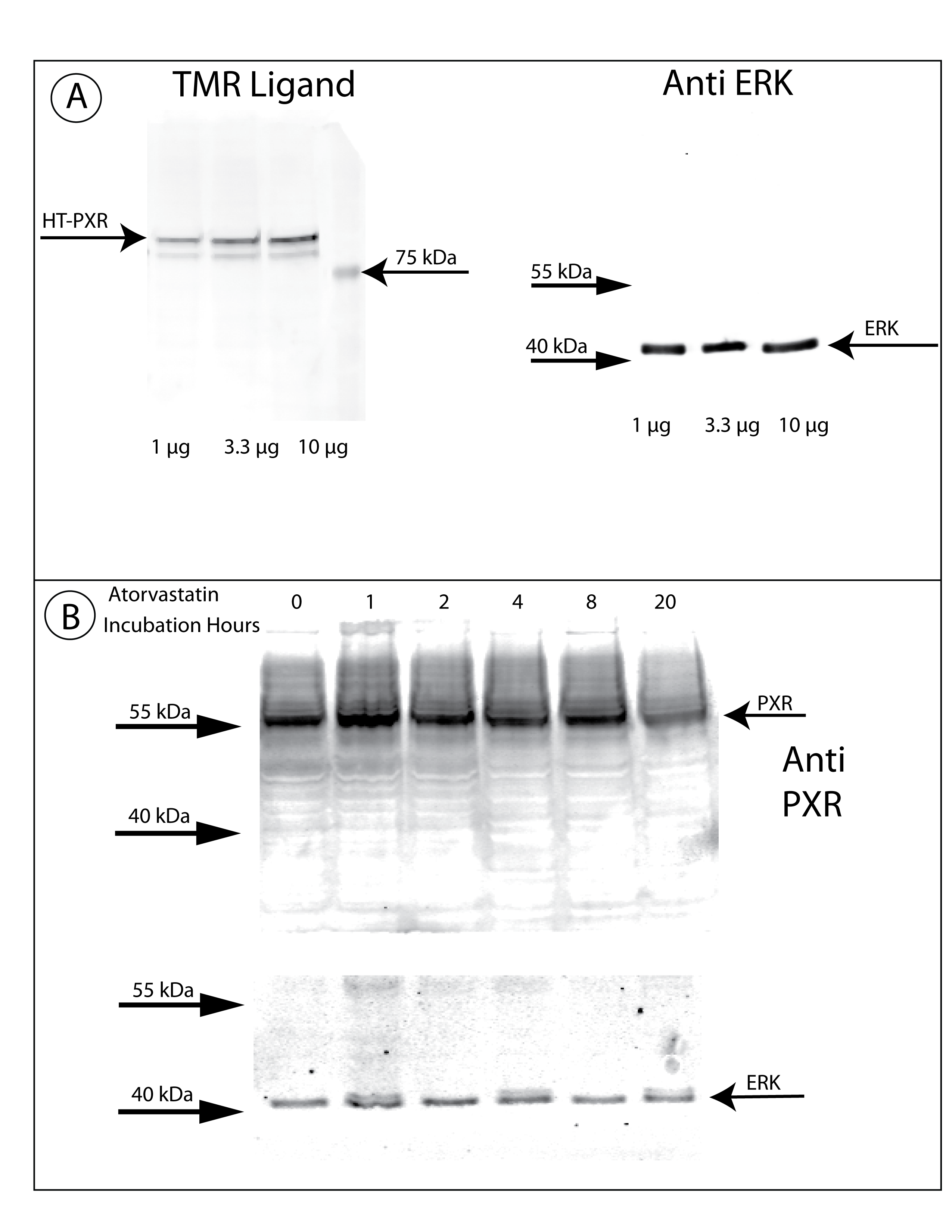

Supplement: S2 Fig — A. Complete blot of the expression of HT-PXR shown in Fig 6B and the complete Erk control blot. B. Complete blot of endogenous PXR levels in response to atorvastatin shown in Fig 6C and the blot of the Erk control. (TIF) [file pone.0138097.s002.tif]
